# Improved Survival with Therapeutic Hypothermia after Cardiac Arrest with Cold Saline and Surfacing Cooling: Keep It Simple

**DOI:** 10.1155/2011/395813

**Published:** 2011-04-12

**Authors:** Cristina Granja, Pedro Ferreira, Orquídea Ribeiro, João Pina

**Affiliations:** ^1^Department of Emergency and Intensive Care Medicine, Hospital Pedro Hispano, 4450 Matosinhos, Portugal; ^2^Department of Biostatistics and Medical Informatics, Faculty of Medicine of Porto, University of Porto, 4200 Porto, Portugal

## Abstract

*Aim*. To evaluate whether the introduction of a therapeutic hypothermia (TH) protocol consisting of cold saline infusion and surface cooling would be effective in targeting mild therapeutic hypothermia (32–34°C). Additionally, to evaluate if TH would improve survival after cardiac arrest. *Design*. Before-after design. *Setting*. General Intensive Care Unit (ICU) at an urban general hospital with 470 beds. *Patients and Methods*. Patients admitted in the ICU after cardiac arrest between 2004 and 2009 were included. Effectiveness of the TH protocol to achieve the targeted temperature was evaluated. Hospital mortality was compared before (October 2004–March 2006) and after (April 2006–September 2009) the protocol implementation. *Results*. Hundred and thirty patients were included, 75 patients were not submitted to TH (before TH group), and 55 were submitted to TH (TH group). There were no significant differences concerning baseline, ICU, and cardiac arrest characteristics between both groups. There was a significant reduction in hospital mortality from 61% (*n* = 46) in the before TH group to 40% (*n* = 22) in the TH group. *Conclusion*. Our protocol consisting of cold saline infusion and surface cooling might be effective in inducing and maintaining mild therapeutic hypothermia. TH achieved with this protocol was associated with a significant reduction in hospital mortality.

## 1. Introduction

The outcome after cardiac arrest is still poor [1], with only 7% to 30% of the patients being discharged from hospital with good neurologic outcome [1]. Therapeutic hypothermia (TH) can improve survival and the neurological outcome [2, 3] after cardiac arrest. Previous and current guidelines recommend TH in comatose survivors of cardiac arrest associated with nonshockable rhythms as well as shockable rhythms, acknowledging, however, the lower level of evidence for use after cardiac arrest from non-shockable rhythms [4, 5]. The effect of hypothermia on the neurological outcome would seem to be most beneficial when the treatment is initiated as early as possible after restoration of spontaneous circulation (ROSC) and maintained for 12–24 h [6]. A recent metanalysis [1, 7] and a revision of the literature [8] have confirmed the benefit of the TH even outside the scope of randomized controlled trials. While several methods of cooling are currently applied [6–14], there is no proof of superiority of any cooling method above others, and there are currently no formal cost-benefit analyses [1]. Surface cooling is generally considered the least expensive and is probably the most widely used [15–17]. A recent study demonstrated that intravenous cold saline infusion combined with ice packs was effective in inducing and maintaining therapeutic hypothermia, with good temperature control. However, this study did not analyze the benefit of TH regarding mortality [17]. The aim of the present study was to further evaluate whether the introduction of a TH protocol consisting of cold saline infusion and surface cooling would be effective in targeting and maintaining mild therapeutic hypothermia (32–34°C) during the first 12 to 24 hours after cardiac arrest and, secondarily, to evaluate if TH would improve survival after cardiac arrest.

## 2. Patients and Methods

The study protocol was approved by the local ethics committee. All patients admitted in the intensive care unit (ICU) after cardiac arrest between 2004 and 2009 were included. Effectiveness of the TH protocol to achieve the targeted temperature was evaluated. Hospital mortality was compared before (2004–2006)—before TH group—and after (2007–2009) the protocol implementation—TH group. Neurological outcome was defined six months after ICU discharge during the ICU follow-up consultation, according to the Pittsburgh cerebral performance category (CPC) [18]—CPC 1 and 2 were classified as a favorable neurological outcome, whereas CPC 3, 4, and 5 were regarded as an unfavorable outcome.

## 3. Therapeutic Hypothermia (TH) Protocol

**Inclusion Criteria**

All patients admitted in the ICU after cardiac arrest were included, independently of the cardiac arrest rhythm, cardiac arrest location (in or out of hospital), witnessed or nonwitnessed cardiac arrest.

**Exclusion Criteria**

**Figure 1:**
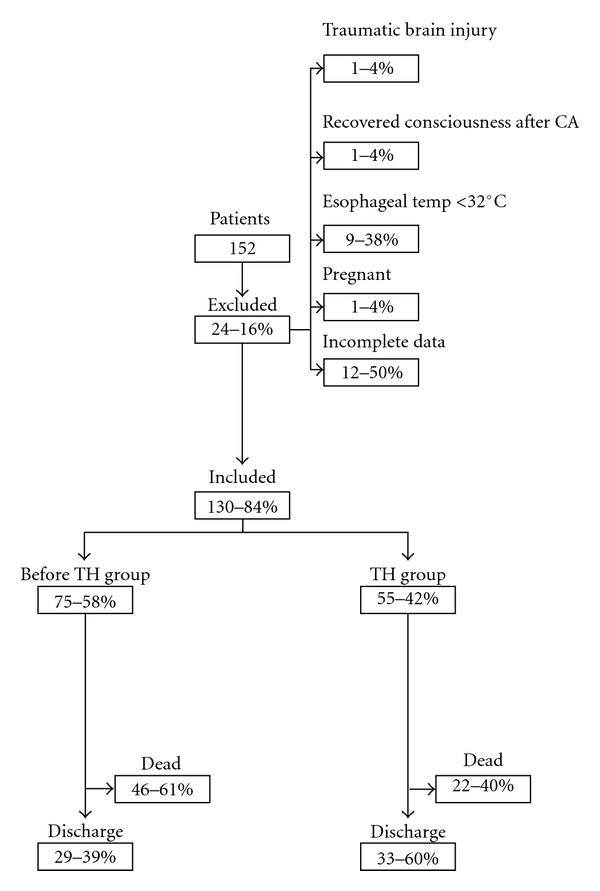
Patients admitted and excluded from the study. Mortality and survival rates.

Patients with less than 18 years old (*n* = 0); those that at admission presented with an esophageal temperature of less than 32°C (*n* = 9); those presenting the following conditions: traumatic brain injury (*n* = 1), pregnancy (*n* = 1), *status epilepthicus *(*n* = 0), persistent and refractory hypotension (systolic blood pressure under 80 mmHg) (*n* = 0), and bleeding diatheses (*n* = 0). Patients that presented incomplete data in the clinical files (*n* = 12), and those who recovered consciousness immediately after cardiac arrest (*n* = 1) were also excluded—Figure 1.

The goal of the TH was to achieve a temperature of 32°C to 34°C as soon as possible after ICU admission and maintain it for a minimum of 12 hrs and a maximum of 24 hrs. All patients were sedated with continuous intravenous infusion of propofol or midazolam plus fentanyl, neuromuscular blockade was maintained with atracurium or rocuronium and they all were mechanically ventilated via endotracheal tubes. Neuromuscular blockade was tested using a train-of-four. Immediately after ICU admission, they were given an intravenous bolus of saline 30 mL/kg at 4°C. Central body temperature was continuously monitored via an esophageal probe. During the time of TH, patients were always without blankets on. To maintain TH, the following successive measures were taken: ice packs were applied in the groins, axillae and along the neck and were periodically changed to maintain low temperature, instillation of cold saline through the nasogastric tube and bladder tube, spray of cold water throughout the body surface intermittently, and continuous cool air through a fan was also included. Efforts to cool the patient were temporarily suspended if the temperature dropped to less than 32°C. Patients were allowed to rewarm passively, and no patient was actively re-warmed.Registered times included the following: time from admission at ICU to reach hypothermia, time of duration of hypothermia, and time to rewarm. Data were collected retrospectively from the clinical files of patients. Descriptive analyses of background variables (gender, age), ICU variables (reason for admission in cardiac and noncardiac, simplified acute physiology score (SAPS II), length of ICU stay (LOS), and ventilation days) and cardiac arrest variables (cardiac arrest cause, rhythm, and duration) were undertaken. Categorical variables were described as absolute frequencies (*n*) and relative frequencies (%); median and percentiles were used for continuous variables. Pearson Chi-square test and Mann-Whitney test were used for comparisons. Statistical significance was considered at *P* < .05. SPSS 17.0 was used for statistical analysis.

## 4. Results

The study included a total of 130 patients, 75 patients from the before TH group and 55 patients from the TH group. Baseline, ICU, cardiac arrest characteristics for the study population and mortality are given in Table 1. Mean time of induction of hypothermia was 4 hours (SD   ±   2,25 h). Mean duration of hypothermia was 15 hours (SD ± 4,11 h). Mean time for passive re-warming was 5 h (SD ± 2,33 h).

ICU and hospital mortality in both groups are shown in Table 1. Hospital mortality was significantly lower in the TH group: 46 patients from the TH group and 29 patients from the before TH group were discharged alive from hospital, which means a 21% reduction in hospital mortality. At six months after ICU discharge CPC was evaluated in 56 patients (28 from the TH group and 28 from the before TH group). There were no significant differences on the CPC between both groups at six months: 26 patients from the TH group and 21 from the before TH group presented favorable neurologic outcome.

Adverse events that might be due to TH included ventilator associated pneumonia in two patients and one ventricular fibrillation in one patient that recovered without sequelae.

## 5. Discussion

Main findings from this study included: first, our protocol, similar to others that described surface cooling [11, 17], was effective in achieving and maintaining TH in patients after cardiac arrest; second, a significant reduction of 21% in hospital mortality was observed in the TH group.

These findings are important as at the present time there is no proof of superiority of any cooling method above others, and there are currently no formal cost-benefit analyses [1]. This is even more important as the recent published ERC/ILCOR guidelines on cardiopulmonary resuscitation recommend TH for both shockable and non-shockable rhythms [5]. Surface cooling is generally considered the least expensive and is probably the most widely used: as thus, it is important to demonstrate the feasibility and the benefit of this least expensive method. We acknowledge that we did not perform any cost analysis study, but others have described surface saline infusion and surface cooling as the least expensive method [15]. Previous studies have described that induction of TH with iced saline is feasible, mainly in the prehospital setting; however, maintenance of TH may be more difficult to achieve [7] our protocol was effective not only in achieving but also in maintaining TH. 

Our findings are not in agreement with previous studies: Kim et al. [19] suggested that passive cooling was ineffective compared with active cooling. However, the study by Kim et al. included only 17 patients, and passive cooling consisted of infusion of 4°C normal saline, use of fans, leaving the patients uncovered and lowering the ambient room temperature. We think that our protocol was more effective because it also included ice packs applied in the groins, axillae, and along the neck that were periodically changed to maintain low temperature, plus instillation of cold saline through the nasogastric tube and bladder tube and also intermittent spray of cold water throughout the body surface.

Some authors have described similar protocols as adding extensive work load to the nurse team and for this reason have abandoned it and changed to other protocols [20]. Nurse workload was not evaluated in the present study but our protocol was feasible. 

Other strength from our study is that patients that were spontaneously with temperatures below 32°C at ICU admission were excluded. This is important as spontaneous hypothermia after cardiac arrest has been associated with a worse outcome [21]. Furthermore, mean times for induction and maintenance were similar to those described by other authors [2, 3, 11].

Storm et al. [22] have found not only a benefit on survival but also on CPC at 2 years after cardiac arrest. However, Bro-Jeppesen et al. [23] found a significant improvement on survival and CPC at hospital discharge but did not find any benefit to survivors 6 months after cardiac arrest. We were not able to find this benefit but we acknowledge that we lost patients to follow up and as thus we were not able to find benefit or not. 

Limitations of the present study include: (1) data collected retrospectively, (2) patients lost to follow up, (3) although frozen saline packs were withdrawal if temperature drops below 32°C, there are no records concerning “overcooling”.

In conclusion, our protocol consisting of cold saline infusion and surface cooling might be effective in inducing and maintaining mild therapeutic hypothermia. TH achieved with this protocol was associated with a significant reduction in hospital mortality. This study adds to our knowledge as it demonstrates not only the feasibility of our protocol but also its benefit on survival.

## Figures and Tables

**Table 1:** Demographic, ICU cardiac arrest characteristics, and mortality of all included patients (*n* = 130).

	Total (*n* = 130–100%)		Before TH group (*n* = 75–58%)		TH group (*n* = 55–42%)		*P*
Age, median (P25–P75)	66	(56–75)	66	(58–75)	65	(55–75)	.307^§^
Sex, *n* (%)							
Male	103	(79)	59	(79)	44	(80)	.853*
Female	27	(21)	16	(21)	11	(20)	
Reason for admission, *n* (%)							
Cardiac	69	(53)	40	(53)	29	(53)	.945*
Non cardiac	61	(47)	35	(47)	26	(47)	
SAPS II, median (P25–P75)	53	(41–61)	49	(39–65)	53	(41–58)	.884^§^
Ventilation days, median (P25–P75)	6	(2–10)	6	(3–10)	5	(2–9)	.199^§^
ICU days, median (P25–P75)	7	(3–11)	7	(4–11)	6	(3–9)	.291^§^
CPR duration, median (P25–P75)	10	(7–19)	10	(7–20)	11	(7–19)	.942^§^
CPR rhythm, *n* (%)							
Asystole	75	(58)	43	(57)	32	(58)	.991*
PEA	14	(11)	8	(11)	6	(11)	
VF	41	(32)	24	(32)	17	(31)	
Mortality, *n* (%)							
Alive	62	(48)	29	(39)	33	(60)	.016*
Dead	68	(52)	46	(61)	22	(40)	

TH: therapeutic hypothermia; SAPS II: simplified acute physiologic score; ICU: intensive care unit; CPR: cardiopulmonary resuscitation; PEA: pulseless electrical activity; VF: ventricular fibrilation.

*P*: percentile; *Chi-square test; ^§^Mann-Whitney test.
